# The conceptualisation of cardiometabolic disease policy model in the UK

**DOI:** 10.1186/s12913-024-11559-y

**Published:** 2024-09-13

**Authors:** Septiara Putri, Giorgio Ciminata, Jim Lewsey, Bhautesh Jani, Nicola McMeekin, Claudia Geue

**Affiliations:** 1 Health Economics and Health Technology Assessment (HEHTA), School of Health and Wellbeing, University of Glasgow, Clarice Pears Building, 90 Byres Road, Glasgow, G12 8TB UK; 2 Health Policy and Administration Department, Faculty of Public Health, University of Indonesia, Depok, Indonesia; 3 General Practice and Primary Care, School of Health and Wellbeing, University of Glasgow, Glasgow, UK

**Keywords:** Conceptual model, Policy model, Decision model, Cardiometabolic disease, Health economics

## Abstract

**Background:**

Decision models are increasingly used to inform policy-making processes, and there is a need to improve their credibility. The estimation of health and economic outcomes generated from decision models is influenced by the development process itself. This paper aims to present the conceptual model development process of cardiometabolic disease (CMD) policy models in the UK setting.

**Methods:**

This conceptual model followed the International Society of Pharmacoeconomics and Outcomes Research-Society of Medical Decision Making (ISPOR-SMDM) Modelling Good Research Practices Task Force-2.

**Results:**

First, for the conceptualisation of the problem, the CMD disease staging, progression and current clinical guidelines were summarised, followed by a systematic review of published policy models. We critically appraised policy models such as cardiovascular disease and type 2 diabetes. Key messages from the review emphasised the importance of understanding various determinants influencing model development, including risk factors, model structure, models’ parameters, data utilisation, economic perspective, equality/equity consideration, transparency and validation process. Second, as a sequential process, is model conceptualisation, to determine which modelling types and their attributes best represent the defined problem. Expert opinions, including a clinician and experienced modellers, provided input on the state transition model to ensure the structure is clinically relevant. From this stage, the consideration and agreement to establish a disease state in a state transition model was discussed.

**Conclusion:**

This conceptual model serves as a basis for representing the systematic process for structuring a CMD policy model to enhance its transparency and credibility.

**Supplementary Information:**

The online version contains supplementary material available at 10.1186/s12913-024-11559-y.

## Background

Cardiometabolic disease (CMD) is an umbrella term encompassing a range of chronic, co-occurring conditions, including cardiovascular disease (CVD), diabetes mellitus, chronic renal failure, hypertension, and stroke [1–3]. The aetiology of CMD is often attributed to shared and frequently co-occurring risk factors, such as dyslipidaemia, obesity, and hypertension [2].

CMDs are a leading cause of mortality and morbidity globally, and they impose a significant economic burden on healthcare systems [4, 5]. In the United Kingdom, approximately 4.7 million people are currently living with diabetes and is projected to rise to over 5.5 million by 2030 [6]. Additionally, an estimated 850,000 individuals remain undiagnosed, further complicating efforts to manage the disease effectively [7]. CVD also poses a substantial public health challenge in the UK, affecting approximately 7.6 million people, including around 4 million men and 3.6 million women who are living with heart and circulatory diseases [8].

Policies for early and timely prevention of CMD are crucial for improving cardiometabolic health. Over several years, modelling techniques have been increasingly applied to assist decision-makers in considering and evaluating public health strategies. A model, particularly in health economics terms, is defined as a structured approach that typically involves the use of mathematical and statistical techniques to simulate the natural history of disease and the impact of particular interventions, leading to the estimation of health outcomes, cost, and cost-effectiveness [9]. The decision analytic model plays a vital role in evaluating these strategies by predicting health and economic outcomes, facilitating scenario analysis, prognosis, and generalisability concerns [10–12]. We therefore propose the development of a CMD policy model, tailored specifically to the UK context.

While decision models are increasingly used to inform the policy-making processes, there is a need to improve their credibility [13]. The development process itself influences the estimation of health and economic outcomes generated from the decision models. An appropriate decision model requires more than mathematical operationalisation alone; it demands understanding complex real-world systems and translating these complexities into credible conceptual structures [14]. This understanding is supported by model structuring, which is pivotal in the decision analytic model. Model structuring involves defining the model’s framework and components, and ensuring all relevant elements such as disease pathways, risk factors, and potential interventions are considered and accurately represented. Once the structure is established, the model can be populated with data and parameters. A conceptual model can illustrate these overall processes [13, 15].

In the health economic model, the transition from conceptual model frameworks to practical model structuring is also crucial for producing reliable and actionable insight. Within the conceptual process, focusing on model structure is important. This involves choosing the appropriate type of model, integrating data, and dealing with uncertainties. It is where abstract ideas from the conceptual model are translated into a quantifiable model. This ensures that the policy model is not only theoretically sound but also practical and relevant for guiding healthcare decision-making [16].

Developing an appropriate conceptual model and specifying key model structure offers several advantages, including enhancing understanding of the decision problem, ensuring alignment with policy objectives, and supporting stakeholder engagement. Conceptual models are instrumental in fostering consensus on problem definition and guiding the development of the model structure [15, 17, 18].

As an initial stage, the conceptual model will be used to guide our CMD policy model development. The CMD policy model is intended to be applied further to estimate costs (e.g., healthcare costs) and outcomes (e.g., life years and quality-adjusted life years) as well as the cost-effectiveness of CMD prevention strategies. This conceptual model is also aimed to enhance the transparency of the model development process, providing clear documentation and justification for the considerations made during its construction.

## Methods

**Fig. 1:**
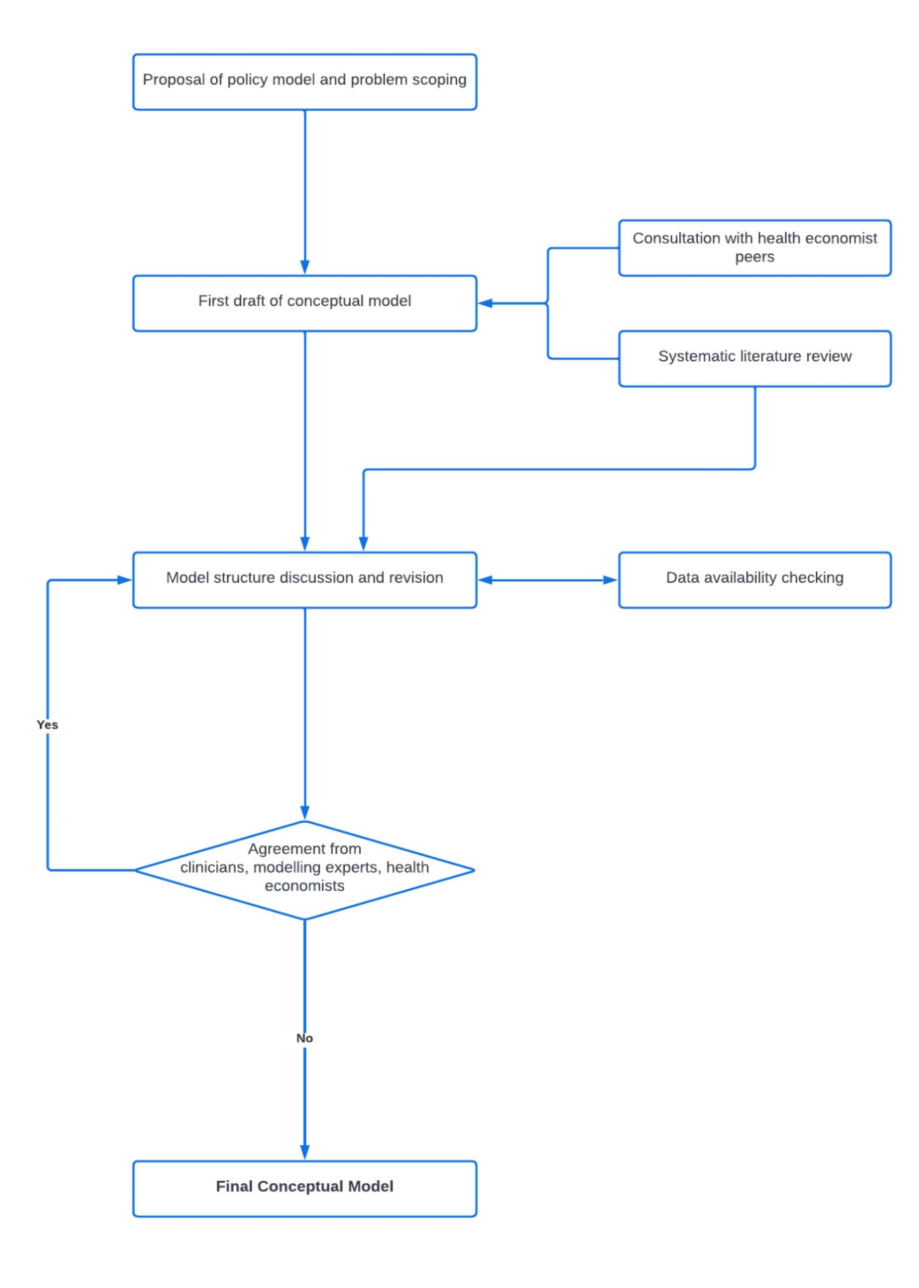
Schematic flow diagram of conceptual model development

This conceptual model followed the International Society of Pharmacoeconomics and Outcomes Research-Society of Medical Decision Making (ISPOR-SMDM) Modelling Good Research Practices Task Force-2 [13]. Two main components of modelling processes are provided in this report. First, is the conceptualisation of the problem, which covers the translation of the healthcare process knowledge into a representation of the problem. Second, as a sequential process, is model conceptualisation, to determine which modelling types and their attributes best represent the defined problem as well as data and parameters used, followed by transparency and validation of the model. The general stage of the conceptual model process is illustrated in Fig. 1.

### Conceptualising the problem

The conceptualisation of the problem requires an understanding of CMD progression and prevention based on clinical and public health guidelines available in the UK. This was followed by conducting a systematic literature review (SLR) of published CMD policy models.

### Systematic literature review (SLR)

Before developing a model, it is crucial to define and elaborate on the decision problem and the significance of modelling within that context. In this study, the scoping of CMD policy model development was initially discussed within a small workgroup (SP, CG, GC, and JL) [19]. This group formulated an initial proposal based on a literature review and potential data sources. Following this, the SLR was conducted to summarise and critically appraise published CMD policy and decision models, with a particular focus on models addressing CVD and T2DM.

The search strategy was applied in multiple databases including MEDLINE (Ovid), EMBASE (Ovid), CINAHL, Google Scholar, and Open Grey, with publication dates restricted from January 1, 2000, to 1st May 2024. A hand search of reference lists from a previous SLR was also conducted using the snowball technique. The review included models that evaluated both long-term health and economic outcomes, with a focus on primordial prevention targeting the entire population or population-based prevention strategies [20].

The review adhered to the Preferred Reporting Items for Systematic Reviews and Meta-Analyses (PRISMA) guidelines [21]. Details of the inclusion and exclusion criteria, as well as the search strategy, were published in the protocol registered on PROSPERO (CRD42022354399) [22]. Data from fully eligible studies were extracted using a standardised table in Excel spreadsheets. The quality of reporting on decision models and economic evaluation studies was assessed by independent reviewers using the Philips et al. checklist [23]. The critical assessment results are presented in a narrative format.

### Conceptualising the Model

#### Expert opinion

The objective of the model development is not to reproduce, but to represent a simplified reality [14]. The model development process should reflect the reality that represents the decision problem. To accommodate this, the role of clinical experts is important to meet contextual relevance [13].

This stage highlights the significance of collaboration between clinical experts and experienced modellers to achieve consensus on model structure. The expert group, consisting of a clinician, two health economists, and a medical statistician, provided diverse and valuable perspectives that contributed to the refinement of the model. The clinician’s input was particularly crucial in ensuring that the model remained aligned with clinical practice and relevant to real-world applications. The proposed conceptual model draft (Additional information 1) was presented to the group, and informal feedback was gathered during the presentation.

After engaging in informal consultations with these experts (2–3 meetings), the conceptual model and model structure were revised to better reflect clinical realities and to enhance its overall validity.

## Results

### Understanding disease progression

The complexity of CMD arises from the interconnection of numerous risk factors (cardiometabolic syndrome (CMS)), including insulin resistance, dyslipidaemia, hypertension, and obesity, as well as comorbidities such as infections, gastrointestinal disease, and kidney disease [1–3]. Without intervention, this can further develop into more severe conditions such as CVD and T2DM [24].

The progression of CMD typically begins with insulin resistance, which may lead to metabolic syndrome or ‘pre-diabetes’. As CMS progresses, the body’s ability to respond to insulin diminishes, compelling the pancreas to compensate by producing higher levels of insulin. However, over time, this compensatory mechanism becomes insufficient, leading to impaired glucose tolerance (IGT) and, ultimately, the onset of T2DM. CMS also doubles the risk of CVD contributing to the rising incidence of heart attacks, strokes, and coronary artery disease. The interplay of insulin resistance, dyslipidaemia, hypertension, and chronic inflammation in CMS accelerates atherosclerosis by promoting endothelial dysfunction, oxidative stress, and plaque formation. This process narrows the arteries, increasing the likelihood of myocardial infarction (MI) and stroke [2, 24–26].

The identification and diagnosis of cardiometabolic syndrome (CMS), which encompasses a cluster of these risk factors, are based on guidelines from several authoritative sources. These include the World Health Organization (WHO) [27], the European Group for the Study of Insulin Resistance (EGIR) [28], the International Diabetes Federation (IDF) [29], the National Cholesterol Education Program Adult Treatment Panel III (NCEP ATP III) [30], the National Heart, Lung, and Blood Institute/American Heart Association (NHLBI/AHA) [31]. Several recent recommendations and guidelines for CMD staging have also been introduced [3, 32, 33]. Based on those guidelines, the general CMD staging system is summarised in Table 1.

**Table 1:** The cardiometabolic disease staging system (CMDS)

Stage	Descriptor	Criteria
**Stage 0**	Metabolically Healthy	No risk factors
**Stage 1**	One or two risks factor	Have one or two of the following risk factors: a. high waist circumference b. elevated blood pressure or on anti-hypertensive medication c. reduced serum HDL cholesterol or on medication d. elevated fasting serum triglycerides or on medication.
**Stage 2**	Metabolic syndrome or prediabetes	Have only one of the following three conditions in isolation a. Metabolic Syndrome based on three or more of four risk factors. b. Impaired Fasting Glucose (IFG) c. Impaired Glucose Tolerance (IGT)
**Stage 3**	Metabolic syndrome + prediabetes	Have any two of the following three conditions: a. Metabolic Syndrome b. IFG c. IGT
**Stage 4**	T2DM and/or CVD	Have Type 2 Diabetes Mellitus (T2DM) and/or cardiovascular disease (CVD): a. T2DM b. CVD

Note: This is general staging. Each clinical guideline may vary slightly in defining staging criteria (risk factors measurement based on gender or consensus standard)

The CMD prevention and treatment guidelines are still in the development stage in the UK [34]. A recent screening strategy has also recently been proposed [35]. However, the diagnosis, prevention, and treatment guidelines for CVD and T2DM have been published by The National Institute of Care and Excellence (NICE) as well as the Scottish Intercollegiate Guidelines Network (SIGN) [36]. Physical activity, dietary recommendations, behavioural changes, and other primordial preventive policies are covered in the guidelines and recommendations. Risk assessment tools such as QRISK3 are used for people without CVD aged between 25–84 years old. Further, lifestyle advice and drug treatment for HbA1c management level for people with T2DM are elaborated in these guidelines. It is evident that these guidelines are continuously evolving based on the latest evidence and are aimed at addressing the prevention and management of CMD in the population [37–39].

Decision modelling which accommodates cost-effectiveness analysis is also available for guidelines in the UK, for instance, existing CVD models simulate several CVD prevention strategies targeting the whole population [38]. Similarly, diabetes models have been used to simulate the prevention and treatment strategies for diagnosed T2DM patients [37]. From these available models and an understanding of cardiometabolic disease staging, a model can be constructed that represents cardiometabolic events (i.e., including both CVD and T2DM).

### Systematic literature review

#### Model types and structure

A total of 44 articles were retrieved for this review. Detailed results and discussions are available in our systematic review published elsewhere [20]. One of the clear advantages of modelling is the capability to estimate and simulate long-term disease progression and the impact of an intervention, which complements RCTs [9, 40]. Our review established that models were either state transition cohort approach (Markov-based cohort) or individual level (microsimulation) approaches, with different perspectives chosen, costs incurred, and sensitivity analysis performed (Additional information 2). The choice between these approaches depends on the study objectives, policy questions, and data availability.

Cohort simulations are advantageous for their efficiency and generalisability but are limited by their inability to account for individual variability, lack of precision, potential for ecological fallacy, and challenges in modelling complex interactions. In contrast, individual-level simulations offer greater granularity and personalised insights, capturing heterogeneity and specific outcomes, but they require extensive data, are resource-intensive, may involve significant uncertainty, and can be less interpretable and generalisable [41–43].

In state-transition models, disease states are defined by clinical guidelines and the natural progression of the disease. The structure of models represented either T2DM or CVD disease progression, and some studies include both of those conditions either as an additional state or as a comorbidity [20]. Although the states represent the final stages in CMD staging, there is a need to include states that are likely to be clinically important in the future, for example, specify complication state for T2DM, or MI and stroke states to represent CVD events.

#### Risk factors

Risk factors are attributes, characteristics, or exposures that increase the likelihood of developing a disease or health condition [44]. The relationship between risk factors and outcomes is essential for predicting the impact of interventions, understanding disease progression, and estimating cost-effectiveness analysis. In a health economics model, the use of risk factors is to demonstrate the causal pathways (e.g., unhealthy diet increases T2DM), transition probabilities (influence probability between health states; e.g., from healthy to disease), and outcome (e.g., affecting mortality and morbidity incidence) [12, 23, 43].

**Fig. 2:**
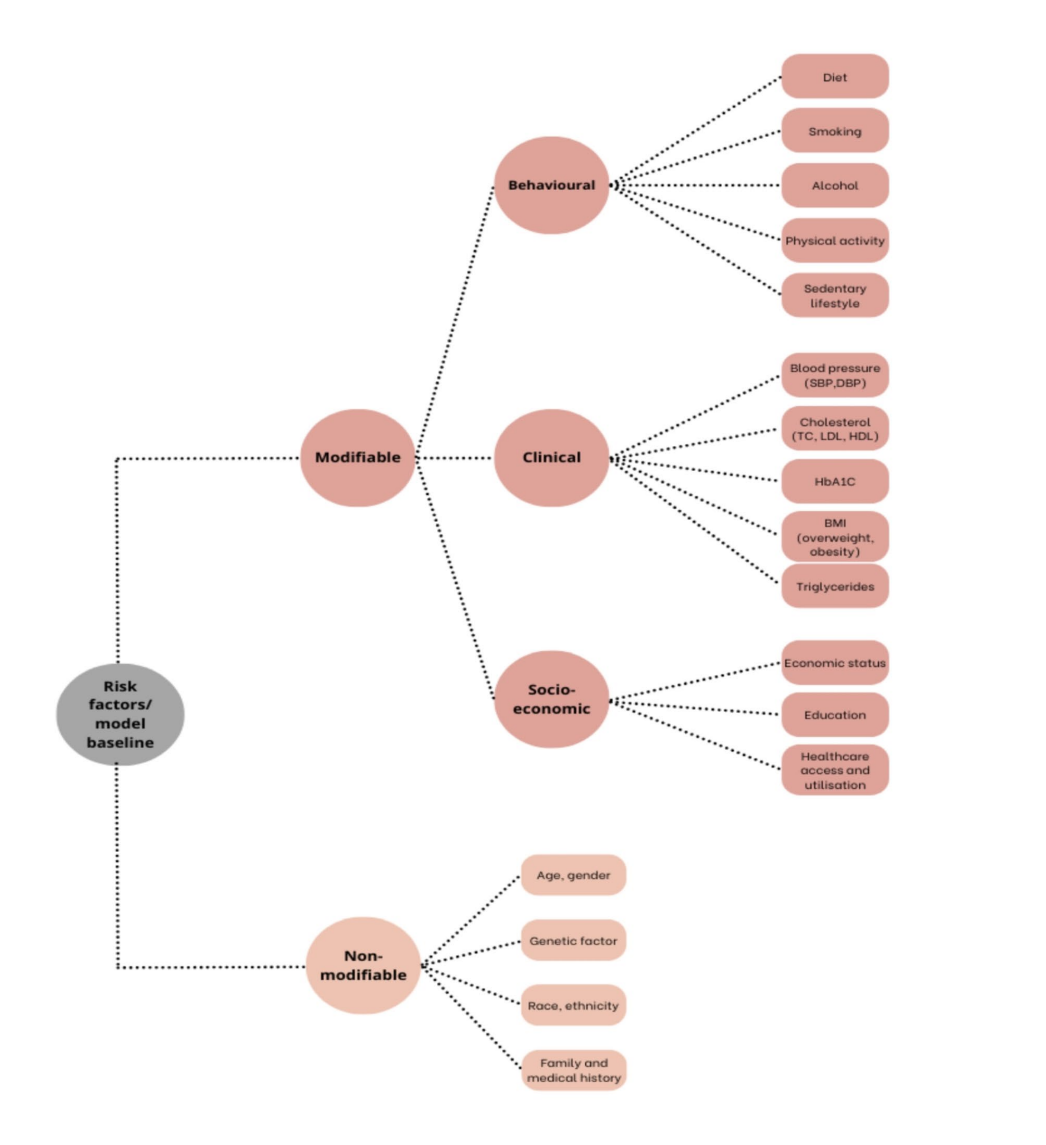
Risk factors included in policy model

Generally, risk factors/covariates can be classified into two main types: modifiable and non-modifiable. Modifiable are further categorised into clinical (BMI, blood pressure), behavioural (diet, smoking) and socio-economic (economic status) (Fig. 2). On the other hand, age, gender, genetic factors, race/ethnicity, and family/medical history are considered non-modifiable risk factors. We included common CMD risk factors in the first draft of our conceptual model.

#### Other model features (costs, outcomes, validation, sensitivity analysis)

Most policy models adopt a healthcare provider perspective, however, incorporating productivity loss from patient and caregiver perspectives can be beneficial to inform decision making. Furthermore, validation tests can test model consistency (e.g., face and internal validation) but need to be transparent in terms of reporting the results and the impact on the modelling. All studies reported that they conducted sensitivity analysis as part of the modelling. These uncertainties are often explored through sensitivity analyses (SA), deterministically and/or probabilistically [20].

Few studies assess the equality and equity assessment, addressing these can help to design mode holistic interventions that balance efficiency with fairness, leading to more socially acceptable and sustainable health policies [20].

The reliability of model appraisals is contingent upon the quality of underlying data. From this SLR, a consistent challenge is the scarcity of representative, locally derived data for model development. Consequently, researchers often rely on assumptions or data from external sources, introducing uncertainty and compromising data validity. While secondary data can be valuable, inconsistencies in data transferability standards and inadequate justifications for data application hinder their effective use. The use of survey and observational data, susceptible to biases, further undermines data quality [45].

To enhance model accuracy and generalisability, the incorporation of appropriate real-world data (RWD) is essential [46]. RWD offers a more representative patient population and treatment outcomes. Nevertheless, RWD presents complexities that should be carefully addressed, including confounding variables, missing data, and lead-time bias, necessitating rigorous methodological considerations to optimise its utility in model development [46, 47].

### Expert opinion

Inputs from a clinician and experts are mostly focused on the proposed model structure and key features of the model since this model is planned to accommodate any further early prevention strategies that can improve cardiometabolic health.

Initially, the risk factors included following the SLR result – see Fig. 2. However, aligned with the clinical guidelines reviewed above, metabolic conditions such as obesity, hypertension and hyperlipidemia/dyslipidaemia are also considered covariates due to their strong correlation with metabolic syndrome [1, 48]. Experts proposed atrial fibrillation (AF) to be included in the health state in the conceptual model, as cardiometabolic risk factors can increase the risk of AF, while AF itself can contribute to the progression of cardiometabolic conditions [49]. There was also an input to re-evaluate the T2DM progression, whether it is adding states with/without diabetes complications before the final state.

The onset of diabetes accelerates the development of atherosclerosis and other CVD risk factors, showing that people with diabetes also have a risk of having CVD [50]. This progression was recommended to be added to the final conceptual model since the first conceptual model did not draw this relationship.

For CVD, we must include the second event (e.g., MI and stroke), and post-CVD event, since there may be differences in terms of utility assessment that could influence the cost-effectiveness results if we plan to conduct further cost-utility analysis (CUA) [51, 52].

Finally, we discussed further with the health economist team that the model should be representative but not too complex, AF remains considered a potential covariate when analysing data. In addition, the structure was presented in an internal meeting with a broad research audience, and several points were discussed. First, the simplicity but representativeness of the model state, considering the feasibility and time needed for analysis and modelling exercise. Second, the possibility to put productivity loss parameters in analysis or sensitivity analyses, therefore the proposed outcome not only healthcare costs but also indirect costs of patients/caregivers perspective. Third, aligned with the findings from SLR, addressing the equality and equity concerns in the model will enrich the further analysis.

### Final conceptual model

**Fig. 3:**
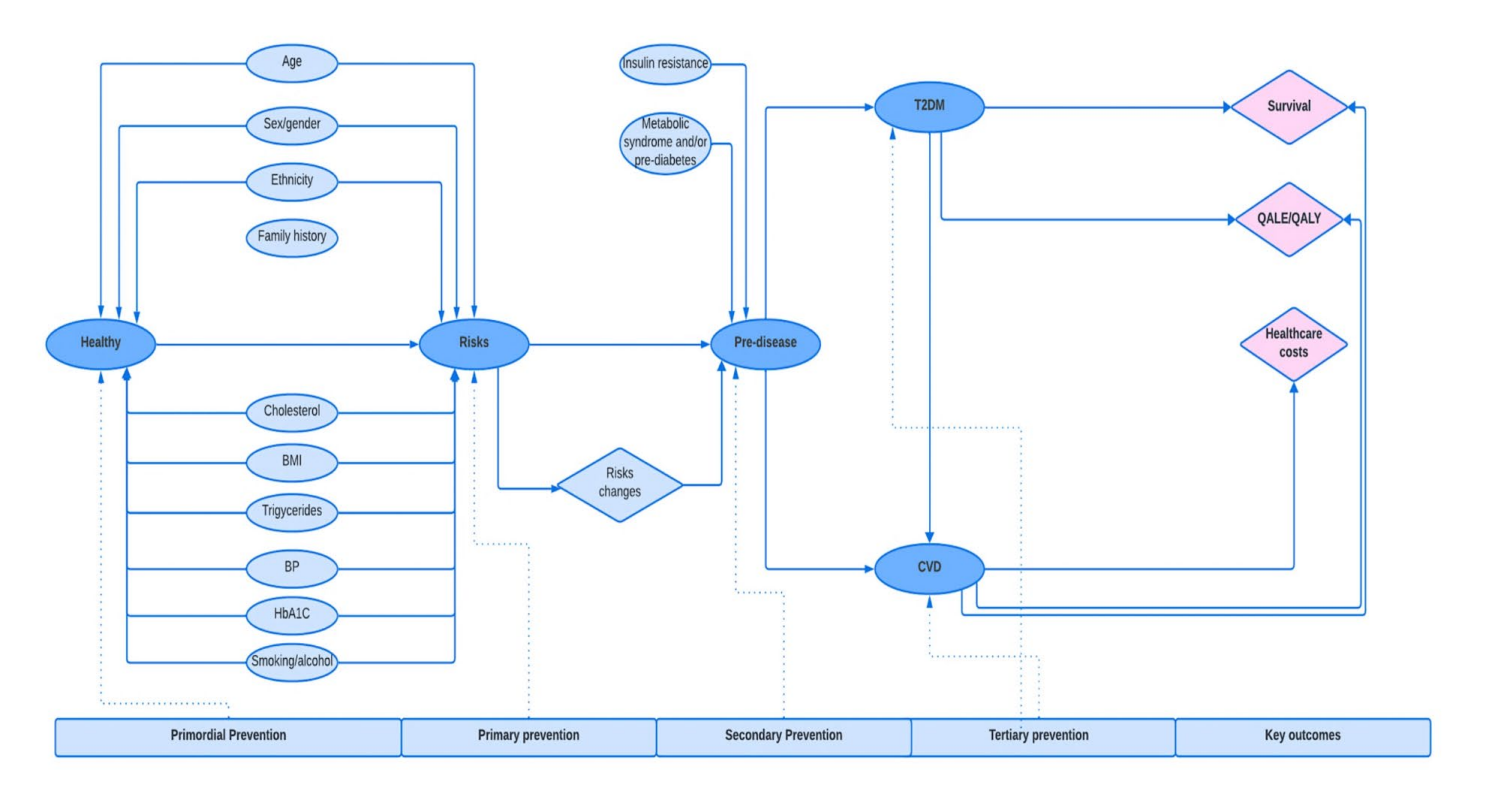
Final conceptual model. T2DM: type 2 diabetes, BMI: body mass index, BP: blood pressure, CVD: cardiovascular disease, QALE: quality-adjusted life expectancy, QALY: quality-adjusted life years

The final conceptual model is illustrated in influence diagram form (Fig. 3). This proposed model will facilitate the improvement of clinical and economic representation of CMD, where metabolic dysfunction conditions could lead to various events including both CVD and T2DM.

To translate this conceptual framework we present a more detailed model structuring stage [16]. A state transition model (STM) will be applied. STM (i.e.: Markov) is well-suited for population health assessment due to several reasons. First, the ability to capture disease progression across different stages within a population by representative health states. Second, this approach is particularly useful when the timing of transitions between health states is important. These models allow for the analysis of long-term outcomes by simulating multiple cycles (e.g., years), capturing the dynamic nature of disease progression (e.g., recurring events such as MI). Third, it can incorporate uncertainty and variability in inputs, allowing for more robust predictions and planning [43, 53, 54]. Specifically, the semi-Markov approach will be applied with several assumptions, such as non-exponential holding time, accounting for time-varying covariates, competing risks, and the ability to integrate with statistical methods such as survival analysis or joint modelling [55, 56].

**Fig. 4:**
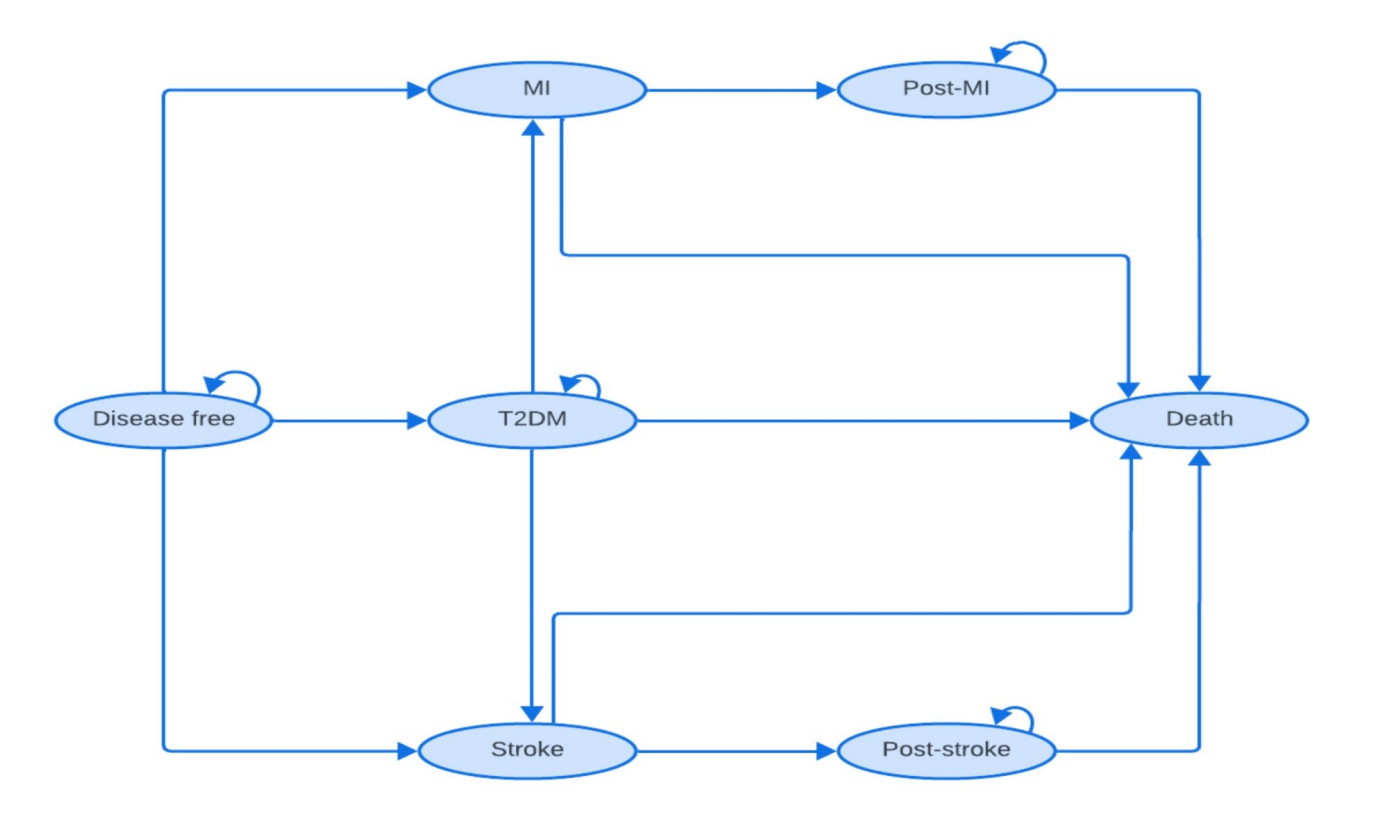
State transition model structure

From the proposed model, the patient population of interest is all adult patients (≥18 years) with no confirmed CMD. The first part specifies patient characteristics when entering the model and specifies them by modifiable and non-modifiable risk factors, as well as deprivation groups. Disease states include disease-free, T2DM, CVD (MI, Stroke), post-CVD (post-MI and post-stroke), and death (Fig. 4).

The Cox multi-state model, as an extension of traditional Cox model will be applied to analyse time to event data where individuals can transition through multiple states over time [57]. This method aligned with the model structured in conceptual model. Transition probabilities between the states are derived and converted from survival rates. The values of the modifiable risk factors will change ‘naturally’ over time and will change the risks of developing CMD when they do so. We will estimate the outcomes including all-cause mortality, disease-related death, life expectancy, QALE/QALY, and lifetime healthcare costs associated with the disease. Sensitivity analysis will be performed both deterministic and probabilistic sensitivity analyses, to handle the uncertainty in the model [43].

Following this conceptual model, for further data analysis, we will utilise Clinical Practice Research Datalink (CPRD) data. CPRD contains data that encompasses 60 million patients, including 16 million currently registered patients [58]. The data records patients anonymously in the electronic health record (EHR) system from general practices (GPs) in England, Scotland, Wales, and the Northern Island on a monthly basis. CPRD includes routine clinical practice information on patients’ demographics, behavioural factors, signs and symptoms, diagnoses, prescriptions, immunisations, referrals, and lifestyle [59]. As well as the strength of a large representative sample size within the UK, CPRD collects data over extended periods, allowing researchers to study the development and progression of diseases over time. In addition, the feasibility of data linkage for hospital and death registries will be beneficial to capturing comprehensive patient’ journeys [58].

## Discussion

It is well-understood that models should be clearly defined and conceptualised before analysis. This paper provides a conceptual model that serves as a foundational framework for developing a policy model that is both appropriate and fit for purpose, by carefully outlining the key components, relationships, and underlying assumptions [13, 14]. It is instrumental in ensuring that the resulting policy model is both theoretically sound and practically effective.

Given adequate reporting quality concluded in the SLR, the findings from our review were deemed reliable and served as a valuable resource for informing the development of the conceptual model. The incorporation of clinical guidelines, the systematic review, and expert input significantly enhanced the model development process. Moreover, particular attention was devoted to the stage of model structuring [16], resulting in a more technically precise and detailed conceptual framework.

Areas requiring further attention include carefully selecting and incorporating relevant parameters, particularly using high-quality routine data to enhance the generalisability of the model’s conclusions [20], which we have tried to address in our final conceptual model. The proposed model structure aligns with established stages of CMD and existing economic evaluation models, demonstrating its consistency with current practices. Based on these findings, no major modifications seem necessary for our conceptual model.

Once the CMD policy model has been developed, it potentially be applied to assess early prevention such as dietary intervention, screening programmes, and preventive medication. Within the analysis, structural sensitivity analysis and model performance evaluation will be conducted following good practice in modelling [23].

To date, the published conceptual models are relatively limited [60–64], and our conceptual model represents a novel contribution to CMD particularly in the health economic modelling area. It extends and enriches existing research by providing a comprehensive and systematic conceptualisation process, by following the good practice for modelling transparency.

We acknowledge several limitations of this conceptual model. First, we asked an experienced clinician and experts to ensure the disease state relevance at a practice level. It is done by gathering input informally during the presentation of the modelling plan. A Delphi process panel with a structured questionnaire potentially improves the process and minimises subjectivity [65]. Second, the structure is trying to cover both T2DM and CVD states that represent major CMD events. Unlike the second event such as the post-CVD event, we did not consider T2DM complications as a second state in the model. Third, we plan to use a healthcare perspective for the model in terms of facilitating further economic analysis. Considering societal perspectives in the model may optimise societal decisions [66]. If any sufficient data is available, we may incorporate this economic perspective in the sensitivity analysis. Furthermore, we are still considering the use of utility value to generate QALY, such as EQ-5D. However, the EQ-5D-5L valuation study remains ongoing for the UK general population [67]. Our solution is to potentially use published EQ-5D-5L for each state (if we conducted a hypothetical public health intervention), or consider QALE as one of the outcomes (without incorporating utility value).

## Conclusions

This paper serves as a first step in representing the systematic process for structuring a CMD policy model in the UK setting. It will be beneficial to enhance our model’s transparency and credibility and also provide insight to a broad audience who are scoping and planning policy models to inform decision-making.

## Electronic supplementary material

Below is the link to the electronic supplementary material.

Supplementary Material 1

Supplementary Material 2

## Data Availability

We do not have any research data outside the submitted manuscript file. All information supporting the findings of this study are available within the paper and its supplementary material.

## Abbreviations

AF: Atrial Fibrillation
CMD: Cardiometabolic disease
CMS: Cardiometabolic syndrome
CPRD: Clinical Practice Research Datalink
CVD: Cardiovascular disease
MI: Myocardial infarction
QALE: Quality-adjusted life expectancy
QALY: Quality-adjusted life years
SLR: Systematic literature review
STM: State transition model
T2DM: Type 2 diabetes mellitus
