# Correction: The conceptualisation of cardiometabolic disease policy model in the UK

**DOI:** 10.1186/s12913-024-11789-0

**Published:** 2024-10-23

**Authors:** Septiara Putri, Giorgio Ciminata, Jim Lewsey, Bhautesh Jani, Nicola McMeekin, Claudia Geue

**Affiliations:** 1 Health Economics and Health Technology Assessment (HEHTA), School of Health and Wellbeing, University of Glasgow, Clarice Pears Building, 90 Byres Road, Glasgow, G12 8TB UK; 2 Health Policy and Administration Department, Faculty of Public Health, University of Indonesia, Depok, Indonesia; 3 General Practice and Primary Care, School of Health and Wellbeing, University of Glasgow, Glasgow, UK

**Correction: BMC Health Serv Res 24, 1060 (2024)**

**https://doi.org/10.1186/s12913-024-11559-y**

Following publication of the original article [1] it was reported that there was an error in Fig. 4. In the original publication there was a missing line between ‘Disease free’ and ‘Death’, as shown in the incorrect figure below:
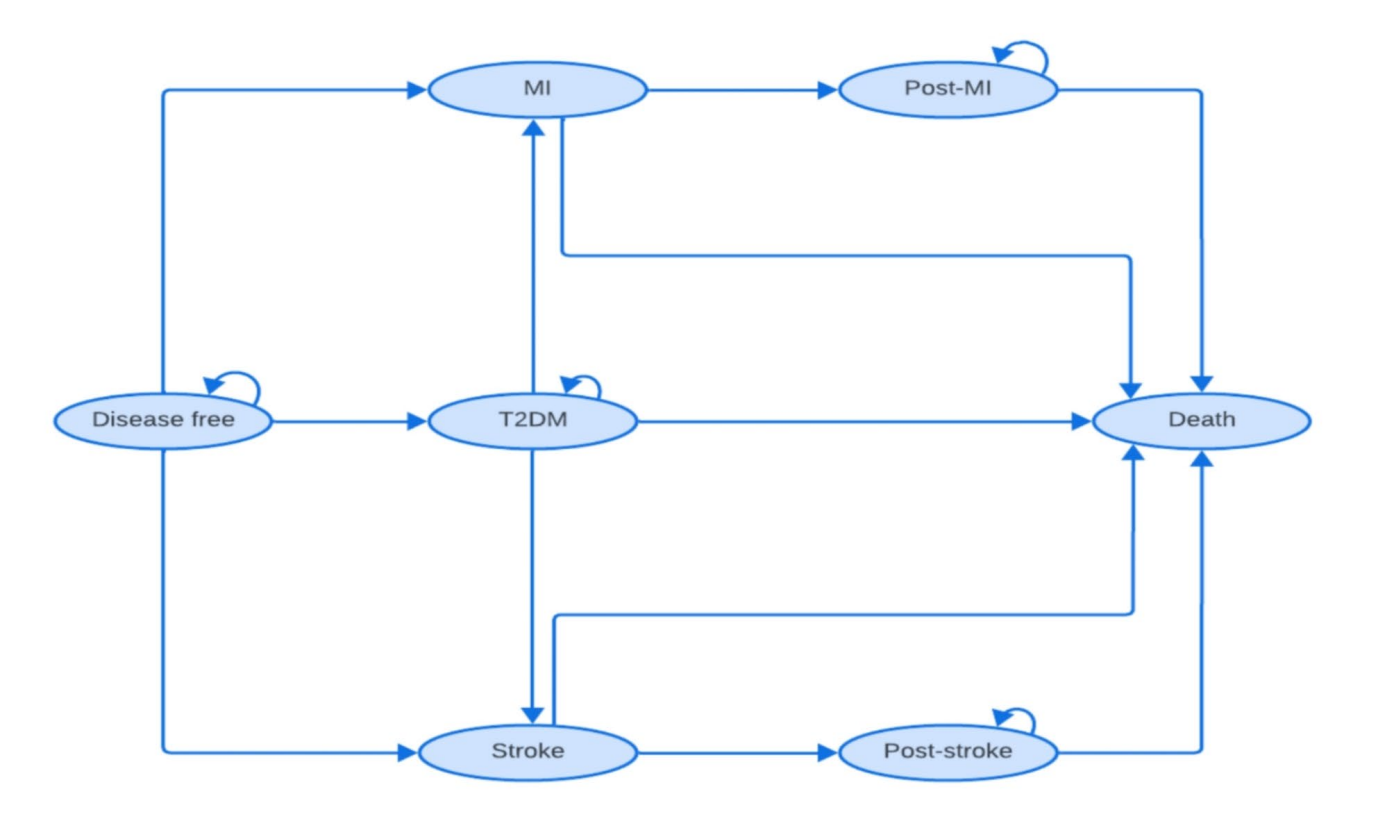


The correct Fig. 4 is as follows:

**Fig. 4:**
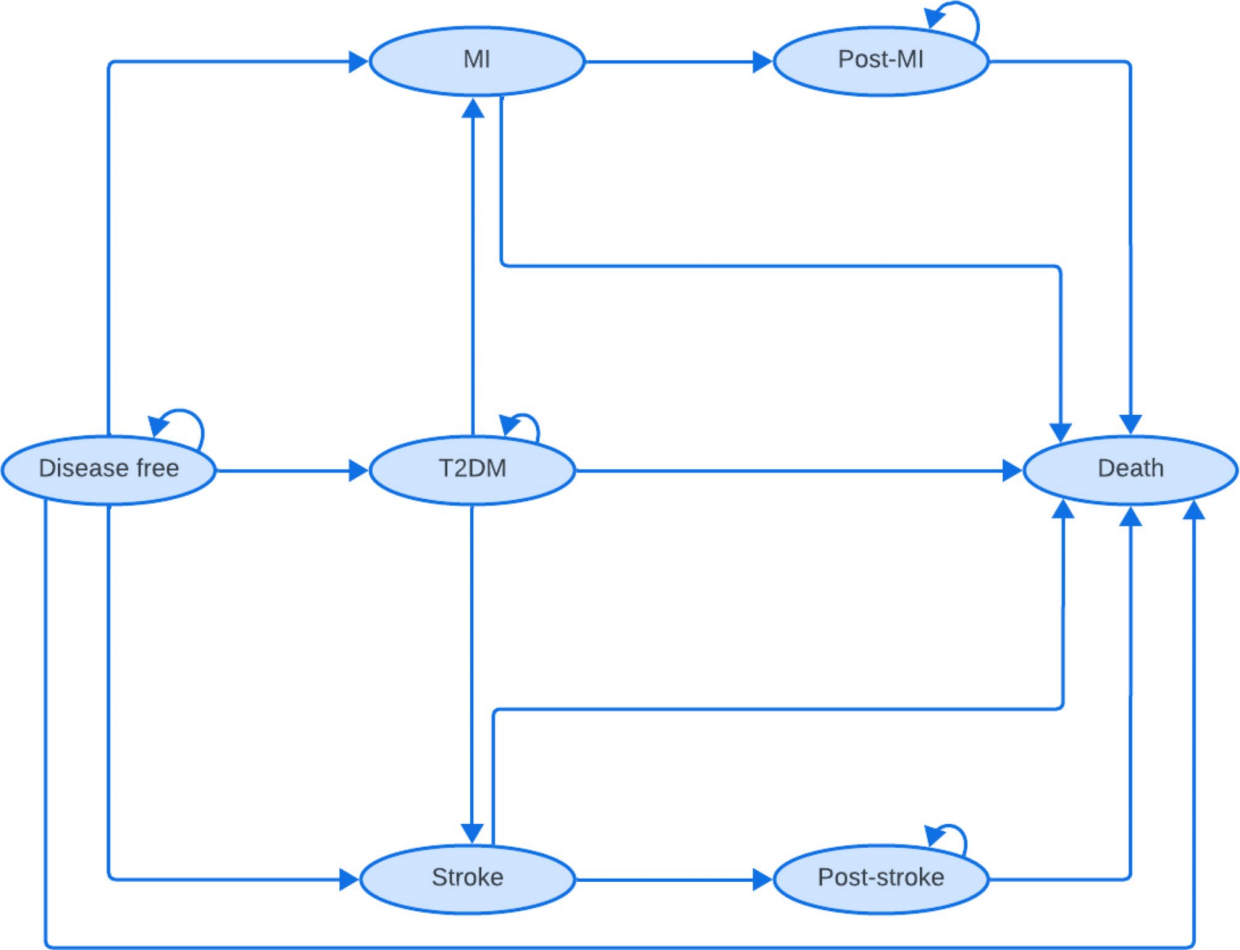
State transition model structure

The original article has been updated.
