# Supplementary material for: The conceptualisation of cardiometabolic disease policy model in the UK
Source: BMC Health Serv Res. 2024 Sep 13;24:1060. doi: 10.1186/s12913-024-11559-y (PMC11396645; doi:10.1186/s12913-024-11559-y)
Supplement: Supplementary file 1 — Supplementary Material 1 [file 12913_2024_11559_MOESM1_ESM.docx]

Additional Information 1. First draft of conceptual model


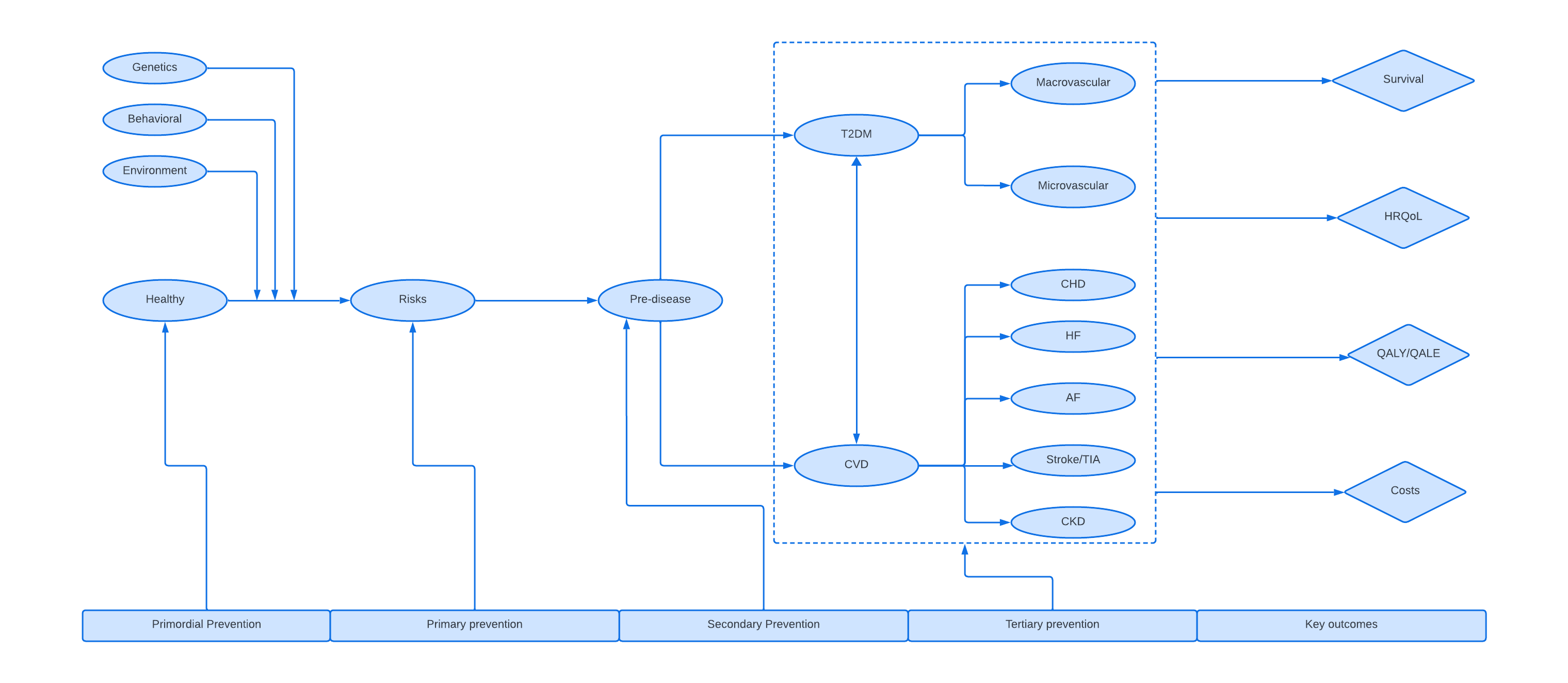


Figure 1. First draft of conceptual model

CVD: cardiovascular disease, T2DM: type 2 diabetes, CHD: chronic heart disease, HF: heart failure, AF: atrial fibrillation, TIA: transient ischemic attack, CKD: chronic kidney disease, HRQoL: health related quality of life, QALE: quality adjusted life expectancy, QALY: quality adjusted life years.
