# Supplementary material for: The conceptualisation of cardiometabolic disease policy model in the UK
Source: BMC Health Serv Res. 2024 Sep 13;24:1060. doi: 10.1186/s12913-024-11559-y (PMC11396645; doi:10.1186/s12913-024-11559-y)
Supplement: Supplementary file 2 — Supplementary Material 2 [file 12913_2024_11559_MOESM2_ESM.docx]

| No. | Author (year) | Country | Model’s name | Policy assessment/ scenarios/evaluation | Perspective | Model types | Time horizon, cycle | Disease states/ measurement | Uncertainty analysis (types) |
| --- | --- | --- | --- | --- | --- | --- | --- | --- | --- |
|  | Dalziel and Segal (2007)^112^ | Australia | - | Cost-effectiveness analysis of 10 nutritional interventions including counselling, diet, lifestyle change, media campaign, nurse check | Societal | Markov | 20 years (except for reduced fat diet scenario), annual | Cardiac model: free, MI, AMI, Major events, stroke, death  Diabetes model: DM, IGT, NGT  F/V model: Success, failure, death  BMI model: Normal, overweight, obese | Yes (DSA) |
|  | Moran et al. (2008)^113^ | China | CHD Policy Model-China | Estimation and assessment of the CHD events based on to demographic changes | N/A | Markov | 30 years, annual | Free CHD, CHD, CHD death, non-CHD death. | Yes (DSA) |
|  | Cobiac et al. (2010)^114^ | Australia | - | Cost-effectiveness analysis of 23 interventions on promoting fruit and vegetable consumption | Health sector | Markov | Lifetime, annual | Healthy, diseased, death (disease), death (other) | Yes (DSA) |
|  | Moran et al. (2010)^115^ | China | CHD Policy Model-China | Estimation of future risk factors on CHD and stroke | N/A | Markov | 20 years, annual | Free CHD, person with CHD, CHD death, non-CHD death. | Yes (DSA) |
|  | Bibbins-Domingo et al. (2010)^116^ | US | CHD Policy Model | Estimation of benefits (rates, costs and cost-effectiveness) of salt reduction intervention | Healthcare | Markov | 10 years, annual | Free CHD, person with CHD, CHD death, non-CHD death. | Yes (DSA) |
|  | Smith-Spangler et al. (2010)^117^ | UK | - | Cost effectiveness analysis of two population strategies for sodium intake reduction | Societal | Markov | Lifetime, annual | Well, MI, stroke, history of MI and stroke | Yes (DSA and PSA) |
|  | Wang et al. (2012)^118^ | US | CHD Policy Model | Estimation of potential health impact and spending of a penny-per-ounce excise nationwide tax policy | Healthcare | Markov | 10 years, annual | Free CHD, person with CHD, CHD death, non-CHD death. | Yes (DSA and PSA) |
|  | Basu et al. (2013)^119^ | US | - | Estimation of health effects and cost-effectiveness SNAP programme | Government | Microsimulation | 10 years, annual | CVD mortality | Yes (DSA and PSA) |
|  | Konfino et al. (2013)^59^ | Argentina | CVD Policy Model-Argentina | Assessment of the impact of sodium reduction policies | N/A | Markov | 10 years, annual | Free CHD, person with CHD, CHD death, non-CHD death. | Yes (DSA) |
|  | Basu et al. (2014)^120^ | India | - | Estimation of the health effect on SSB taxation policy | Government | Microsimulation | 10 years, annual | T2DM incidence | Yes (DSA) |
|  | Collins et al. (2014)^121^ | England | CHD IMPACT Model | Cost-effectiveness analysis of four population health policies on salt intake | Health sector | Cell-based model | N/R | CHD death | Yes (DSA and PSA) |
|  | Mason et al. (2014)^122^ | Tunisia, Syria, Palestine, Turkey | CHD IMPACT Model | Cost-effectiveness analysis of population-based salt reduction policies in four Eastern Mediterranean countries | Public/ private sector, healthcare | Cell-based model | N/R | CHD death | Yes (DSA) |
|  | Lewsey et al. (2015)^54^ | Scotland | Scottish CVD Policy Model | The development of CVD policy model that predicts life expectancy and incorporating socioeconomic deprivation | - | Markov | Potentially lifetime, annual | CVD event free, non-fatal CHD, non-fatal CBVD, fatal CVD, fatal non-CVD, fatal all cause | Yes (DSA and PSA) |
|  | Manyema et al. (2015)^123^ | South Africa | - | Estimation of the effect of 20% SSB tax on the diabetes burden | Healthcare | Markov-multi state life table | 20 years, annual | BMI changes, diabetes | Yes (DSA) |
|  | Wilcox et al. (2015)^124^ | Syria | CHD IMPACT Model | Cost-effectiveness analysis of salt reduction policies | Public/ private sector, healthcare | Cell-based model | 10 years, annual | CHD death | Yes (DSA) |
|  | Collins et al. (2015)^125^ | England | - | Projection of 20 % of sugary drinks duty impact on disease events | Healthcare | Microsimulation | 20 years, annual | Diabetes, stroke, CHD | Yes (PSA) |
|  | Lawson et al. (2016)^55^ | Scotland | Scottish CVD Policy Model | The development of model for conducting economic evaluation | N/A | Markov | Potentially lifetime, annual | CVD event free, non-fatal CHD, non-fatal CBVD, fatal CVD, fatal non-CVD, fatal all cause | Yes (DSA and PSA) |
|  | Sa´nchez-Romero et al. (2016)^57^ | Mexico | CVD Policy Model-Mexico | Projection of SSB tax policies | N/A | Markov | 10 years, annual | No event, CVD event (MI, stroke, angina), death | Yes (DSA and PSA) |
|  | Nghiem et al. (2016)^126^ | New Zealand | - | Estimation of the cost and benefit from dietary sodium interventions | Health system | Markov | Lifetime, annual | CVD free, CHD, stroke, death | Yes (DSA and PSA) |
|  | Veerman et al. (2016)^127^ | Australia | - | Estimation of the effect on 20% SSB tax on health and healthcare expenditure | Health sector | Markov | Lifetime, annual | T2DM, heart disease, stroke | Yes (DSA) |
|  | Wang et al., (2016)^128^ | China | CVD Policy Model-China | Estimation of the effect of population-wide salt restriction in China | Healthcare system payer’s | Markov | Lifetime, annual | CVD free, acute CVD events, chronic CVD states, fatal CHD or stroke, non-CVD death | Yes (DSA) |
|  | Breeze et al. (2017)^129^ | UK | SPHR  Model | Cost effectiveness analysis of different interventions for type 2 diabetes prevention | NHS/PSS | Microsimulation | Lifetime, annual | Metabolic profile, no diabetes, diabetes, complications, CVD, cancer, osteo, depression, mortality | Yes (DSA and PSA) |
|  | Cobiac et al. (2017)^130^ | Australia | - | Estimation of the potential impact of dietary policies | Health sector | Markov-multi state lifetable | Lifetime, annual | Healthy, diseased, death (disease), death (other) | Yes (DSA) |
|  | Pandya et al. (2017)^131^ | US | CVD- PREDICT | Description of the CVD model in detail; and performed model validation analyses | N/A | Microsimulation | Potentially lifetime | Disease free, CHD, stroke, death | N/R |
|  | Choi et al. (2017)^132^ | US | - | Cost-effectiveness analysis of subsidizing FV purchases | Societal | Microsimulation | Lifetime, annual | Demographic status, daily food consumption, disease risk in BMI, diabetes, MI, stroke, lung cancer | Yes (DSA and PSA) |
|  | Mozaffarian et al. (2018)^133^ | US | CVD-PREDICT | Estimation of the health impact and cost-effectiveness in SNAP program | Societal and government | Microsimulation | 5-20 years and lifetime, annual | No CVD, acute CHD, chronic CHD, repeat MI or CVA, acute CVA, chronic CVA, CVD or non-CVD death | Yes (DSA and PSA) |
|  | Riveros et al. (2018)^134^ | Brazil | Adaptation of Scottish CVD Policy Model | Calibration of Brazilian CVD model | N/A | Markov | N/R | CVD event free, non-fatal CHD, non-fatal CBVD, fatal CVD, fatal non-CVD, fatal all cause | - |
|  | Jones (2018)^135^ | Canada | N/A | Estimation of the impact of interventions to reduce high sugar beverages consumptions | Health sector | Markov (multi state lifetable) | 25 years, annual | Healthy, diseased, death (disease), death (other) | Yes (DSA and PSA) |
|  | Schönbach et al. (2018)^136^ | Germany | DYNAMO-HIA | Estimation of health impact of tax on processed meat | N/A | Markov (extend to microsimulation) | 10 years, annual | Prevalence in CHD, diabetes, cancer | Yes (DSA and PSA) |
|  | Huang et al. (2019)^137^ | US | CHD IMPACT model | Estimation of the health impact and cost-effectiveness added sugar labelling on all packaged food and beverages | Healthcare and societal | Cell-based model | 20 years, annual | CHD incidence, stroke incidence, T2DM incidence | Yes (DSA and PSA) |
|  | Salgado et al. (2019)^138^ | Argentina | CVD Policy Model-Argentina | The update Argentina CVD Policy Model | N/A | Markov | Lifetime, annual | CVD free, acute CVD events, chronic CVD states, fatal CHD or stroke, non-CVD death | N/R |
|  | Wilde et al. (2019)^139^ | US | CVD-PREDICT | Estimation of the health impact and cost-effectiveness of a national penny per-ounce SSBs tax | Healthcare and societal | Microsimulation | Lifetime, annual | Disease free, CHD, stroke, death | Yes (DSA and PSA) |
|  | Broeks et al. (2020)^140^ | Netherlands | DYNAMO-HIA | Estimation of the effects of a tax on meat and a subsidy on fruit and vegetables (F&V) consumption | Societal | Markov | 30 years, annual | Healthy, disease, death | Yes (DSA and PSA) |
|  | Lee et al. (2020)^141^ | US | CVD-PREDICT | Estimation of the health impact and cost-effectiveness of three SSBs tax designs | Healthcare, government, societal | Microsimulation | Lifetime, annual | Disease free, CHD, stroke, death | Yes (DSA and PSA) |
|  | Kao et al. (2020)^142^ | Canada | - | Estimation of the health and financial impact of sugary drink tax across different income group | Health system | Markov-multi state life table | Lifetime, annual | Healthy, diseased, death (disease), death (other) | Yes (DSA) |
|  | Liu et al. (2020) ^143^ | US | CVD-PREDICT | Estimation of the health impact and cost-effectiveness of federal restaurant menu calorie labelling policy | Healthcare and societal | Microsimulation | Lifetime, annual | Disease free, CHD, stroke, death | Yes (DSA and PSA) |
|  | Salgado et al. (2020)^144^ | Argentina | CVD Policy Model-Argentina | Estimation of the impact of reducing SSB consumption | N/A | Markov | 10 years, annual | CVD free, acute CVD events, chronic CVD states, fatal CHD or stroke, non-CVD death | Yes (DSA and PSA) |
|  | Marklund et al. (2020)^145^ | Australia | - | Simulate the impact of eliminating trans-fatty acid on the health outcomes | Health sector | Markov | 10 years, annual | Healthy, IHD, post IHD, death | Yes (DSA and PSA) |
|  | Dehmer et al. (2020)^146^ | US | - | Evaluate prospective CVD related sodium reduction targets | Healthcare | Microsimulation | 10 years, annual | Disease free, hypertension, CVD, post-CVD, death | Yes (DSA) |
|  | Shangguan et al. (2021)^147^ | US | CVD-PREDICT | Assessment of the effect of NSSRI (National Salt and Sugar Initiative) sugar reformulation policy | Healthcare and societal | Microsimulation | Lifetime, annual | Sugar intake, acute CVD, diabetes, chronic CVD, CVD or non-CVD death | Yes (DSA and PSA) |
|  | Ikeda et al. (2022)^148^ | Japan | - | Projection of health  and economic benefits of dietary salt reduction policies in Japan | Healthcare | Markov | 10 years, annual | Healthy, chronic  IHD, chronic stroke, death from IHD, death from stroke, and death from other causes | Yes (DSA) |
|  | Marquina et al. (2022)^149^ | Australia | - | Estimation of health and economic burden of new and established CVD; | Societal | Markov | 20 years, annual | No event, CVD, CVD and non-CVD death | Yes (PSA) |
|  | Thomas et al. (2022)^150^ | England | SPHR model | Estimation of health benefits, costs, and equity impact of food advertising across London transport network | NHS/PSS | Microsimulation | Lifetime | Metabolic profile, no diabetes, diabetes, complications, CVD, cancer, osteo, depression, mortality | Yes (DSA and PSA) |
|  | Lou et al. (2023)^151^ | US | CVD Policy Model | Impact assessment of implementing SSB taxes  and FV subsidies on long-term CVD outcomes and  healthcare costs in NYC | Societal | Microsimulation | 10 years, annual | Healthy, CHD, stroke, both CHD and stroke, CVD-related death, and non-CVD-related death | Yes (DSA) |
